# Reduced alternative splicing of estrogen receptor alpha in the endometrium of women with endometriosis

**DOI:** 10.18632/oncotarget.22701

**Published:** 2017-11-27

**Authors:** Ying Zhang, Yong Zhang, Chunbo Zhao, Tiantian Yu, Ye Liu, Weihui Shi, Fengtao Shi, Xinmei Liu, Jianzhong Sheng, Hefeng Huang, Hong Xu

**Affiliations:** ^1^ International Peace Maternity and Child Health Hospital, School of Medicine, Shanghai Jiao Tong University, The China Welfare Institute, Shanghai, China; ^2^ Institute of Embryo-Fetal Original Adult Diseases, School of Medicine, Shanghai Jiao Tong University, Shanghai, China; ^3^ Department of Obstetrics and Gynecology, The Second Affiliated Hospital (Jiande Branch), Medical School of Zhejiang University, The First People's Hospital of Jiande, Hangzhou, China; ^4^ The Key Laboratory of Reproductive Genetics, Ministry of Education (Zhejiang University), Hangzhou, China; ^5^ Department of Pathology and Pathophysiology, School of Medicine, Zhejiang University, Hangzhou, China

**Keywords:** ERα, endometriosis, splice variant, dysmenorrhea

## Abstract

Endometriosis is a condition which involves the presence of uterine stroma and glands outside of the uterine cavity and represents one of the most prevalent disorders of the female reproductive tract. The key symptom of endometriosis is pain, including dysmenorrhea, deep dyspareunia, and chronic pelvic pain. As such, endometriosis has significant economic consequences within the healthcare system and can influence the daily quality of life in affected patients. However, the pathophysiology of this disease and the mechanisms in which this condition generates pain are very unclear. This study, involving 30 women with endometriosis and 28 controls without endometriosis, aimed to investigate relative levels of estrogen receptor alpha (ERα) splice variants in the endometrium of women with and without endometriosis and investigate potential links to the severity of pain. Wild type (wt)-ERα was dominantly expressed in human endometrium while the expression of ERα-del.4, ERα-del.7, and ERα-del.3,4 was significantly reduced in endometriosis patients compared with healthy patients (*p* < 0.05). Furthermore, the relative ratios of wtERα:ERα-del.4, and wtERα:ERα-del.3,4 were associated with the severity of pain in endometriosis patients (*p* < 0.05). Consequently, analyzing differences in the relative levels of four types of ERα splice variant in the endometrium of patients with endometriosis may help in the development of endometriosis-targeted treatment and the development of appropriate therapies.

## INTRODUCTION

Endometriosis, defined as the presence of uterine stroma and glands outside of the uterine cavity, is one of the most prevalent disorders of the female reproductive tract [1]. The key symptom of endometriosis is pain, including dysmenorrhea, deep dyspareunia, and chronic pelvic pain. The burden of endometriosis has significant economic consequences within the healthcare system [2] and can influence the patient's daily quality of life, including sexual activity, emotional interaction, social activity, family relationships, and performance at work [3]. The pathophysiology of this disease, and its mechanisms in which this condition generates pain, are very unclear.

Estrogen has been recognized as an important factor in the pathogenesis of endometriosis [4]. Moreover, the role and interplay of estrogen receptors (both estrogen receptor alpha and beta [ERα and ERβ]), and their associated splice variants, are increasingly being recognized as playing pivotal roles in estrogen-dependent diseases such as breast cancer [5].

ERα is a ligand-dependent transcription factor involved in a wide spectrum of biological processes in both normal and neoplastic tissues. ERα is found on chromosome 6q and encodes for a 595 amino acid protein [6]. Upon binding estrogen (E), ERα dimerizes to promote strong binding of the E:ER complex to estrogen response elements, the specific DNA target of ERα, thus initiating the transcription of target genes [7]. In the absence of E, ERα is thought to be associated with a protein complex, including heat-shock proteins such as HSP-90 [8].

Alternative splicing is an important mechanism underlying transcript variation and is sometimes referred to as exon-skipping in which multiple forms of mRNA are generated from a common pre-mRNA via the differential use of exonic DNA. A growing body of evidence suggests that some ERα splice variants play an important role in both maintaining normal physiology and influencing susceptibility to a variety of diseases, including breast cancer, endometrial cancer and Alzheimer's disease (AD).

The presence of ERα mRNA variants in breast cancer cell lines, as well as tissue from breast cancer patients, has been well established in the literature [9, 10]. Constitutively-active ERα variants could result in the unregulated growth of estrogen-responsive tissue; an effect that would not be interrupted by anti-estrogens. A dominant negative ER with the wild-type ERα variant would complete for anti-estrogen binding, thus diminishing its effects upon tissue [11–13]. Estrogen and some selective ER modulators are known to stimulate proliferation of the endometrium, thus increasing the risk of endometrial carcinoma [14, 15].

While the role of ERs, and their splice variants, in estrogen-dependent disease is becoming more and more accepted, very little is known about the presence of ERα splice variants in the human endometrium. An earlier study identified ERα-Del.4 and ERα-Del.7 (representing ERα splice variants missing exons 4 and 7, respectively) in the human endometrium and also in endometrial carcinoma [16]. However, we know little about the distribution and expression of ERα splice variants in the human endometrium, either with or without endometriosis.

Consequently, our present investigation aimed to identify ERα splice variants in the endometrium and to quantify relative differences in expression in human endometrium with and without endometriosis.

## RESULTS

### Basic clinical information

In total, we recruited 58 patients between September 2015 and December 2015 at the Department of Gynecology and Obstetrics, International Peace Maternity and Child Health Hospital of Shanghai Jiao Tong University (Shanghai, China).

The mean age of patients with and without endometriosis were 34.93 ± 9.25 and 38.75 ± 8.35 years, respectively. We detected significant differences between endometriosis and non-endometriosis groups in terms of dysmenorrhea (*p <* 0.001), parity (*p* = 0.035), and the frequency of sexual activity (*p* = 0.034). However, we failed to observe any statistically significant differences between the two groups in terms of age, body mass index (BMI), blood pressure and heart rate. Further analysis also failed to detect statistically significant differences between the two groups when we considered age at menorrhea, education, occupation, residential status, smoking, alcohol intake, exercise and mode of delivery (Table 1).

**Table 1:** Clinical information of our patient cohort with and without endometriosis

Clinical characteristic	Control (*n* = 28)	Endometriosis (*n* = 30)	*P* value
Age (y)	38.75 ± 9.25	34.93 ± 8.35	0.085
Body mass index (kg/m^2^)	22.49 ± 4.46	21.81 ± 3.03	0.473
Systolic pressure (mmHg)	111.25 ± 12.12	109.57 ± 11.10	0.582
Diastolic blood pressure (mmHg)	76.71 ± 9.63	74.80 ± 9.6	0.455
Heart beats (times/min)	72.18 ± 9.89	70.63 ± 8.99	0.53
Age at menarche (y)	13.86 ± 1.30	14.00 ± 1.23	0.662
Age at first intercourse (y)	23.18 ± 2.14	24.3 ± 4.67	0.312
Pain score	0.53 ± 0.03	5.7 ± 0.21	< 0.001
Highest education (*n*)			
Middle school or less	8	12	Reference
High school	15	14	0.419
University or above	5	4	0.282
Occupation			0.284
Mental status	17	14	
Physical status	11	16	
Salary RMB/m (*n*)			
< 5000	4	5	Reference
5000–20,000	18	17	0.709
> 20000	7	8	0.641
Residence (*n*)			
Urban	17	21	0.457
Rural	11	9	
Smoker (*n*)			
Never	19	20	Reference
Former	7	6	0.749
Current	2	4	0.482
Alcohol use (*n*)			
Less than monthly	13	17	reference
Weekly-monthly	10	6	0.216
Daily-weekly	5	7	0.921
Regular exercise (*n*)			
No	19	17	0.380
Yes	9	13	
Parity (*n*)			
Nulliparous	7	16	0.035
Parous	21	14	
Cesarean delivery (*n*)			
No	14	5	0.094
Yes	7	9	
Vaginal delivery (*n*)			
No	7	9	0.094
Yes	14	5	
Abortion or curettage (*n*)			
No	19	18	0.534
Yes	9	12	
Sexually active frequency (times/month)			
< 5	9	17	0.034
≥ 5	21	11	

### DNA sequencing and mRNA expression

**Figure 1:**
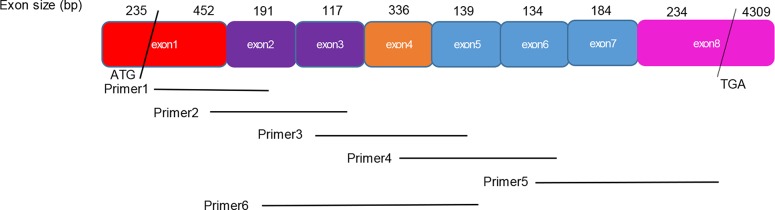
Structure of estrogen receptor alpha (ERα) and primers designed for reverse-transcription polymerase chain reaction (RT-PCR) experiments

**Figure 2:**
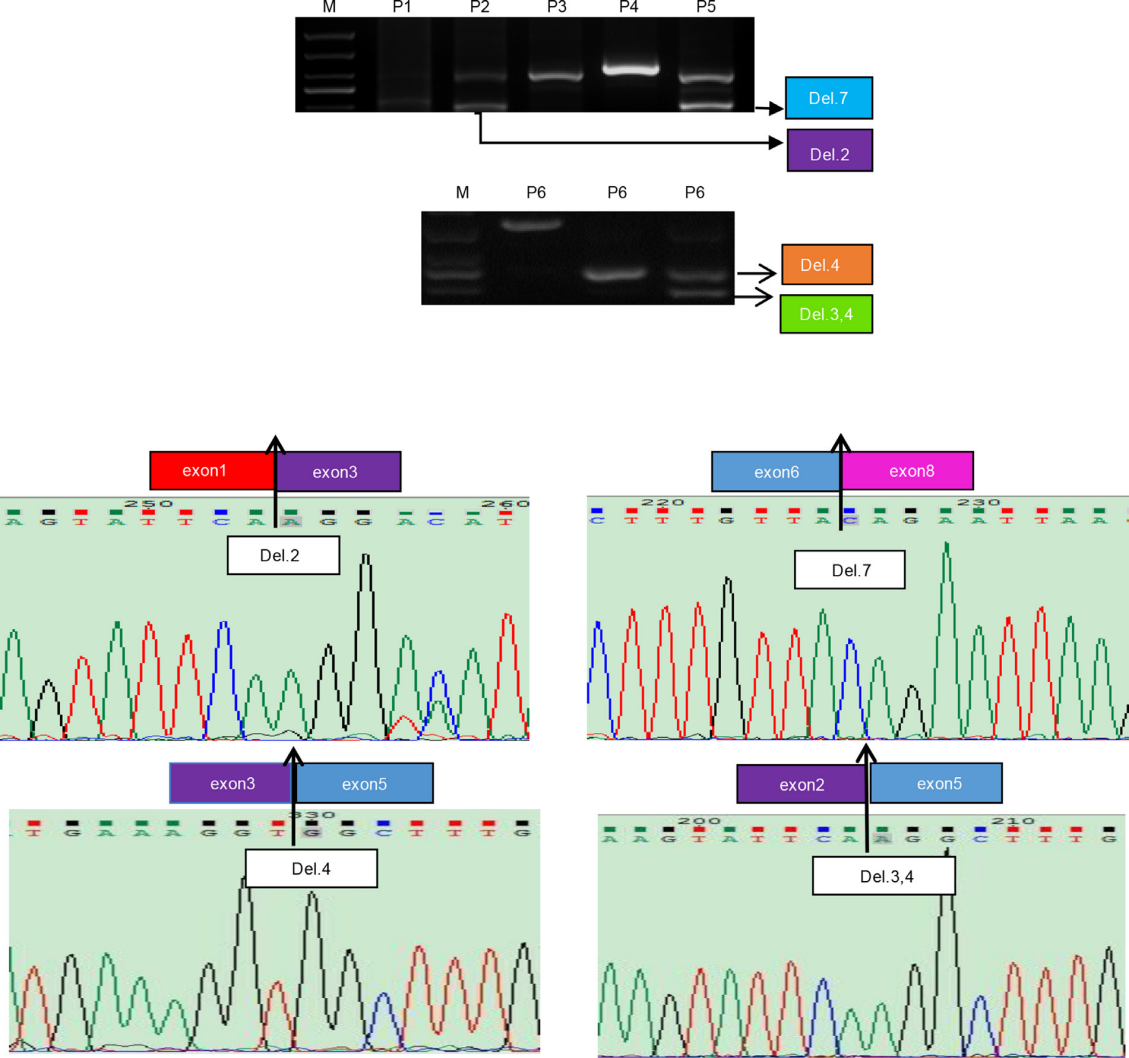
Identification of four estrogen splice variants in the human endometrium Amplified polymerase chain reaction (PCR) products were purified from agarose gels, cloned and then sequenced.

Analysis of ERα mRNA transcripts in samples of human endometrium, both with and without endometriosis, are presented in Figure 1. In addition to wild-type (wt) ERα mRNA, we successfully detected the mRNA of four different types of splice variant in the human endometrium. These represent splice variants lacked exon 2 (Del.2), lacking exon 4 (Del.4), exon 7 (Del.7) and exons 3 and 4 (Figure 2A, 2B).

### Expression of ERα and its splice variants in the human endometrium

**Figure 3:**
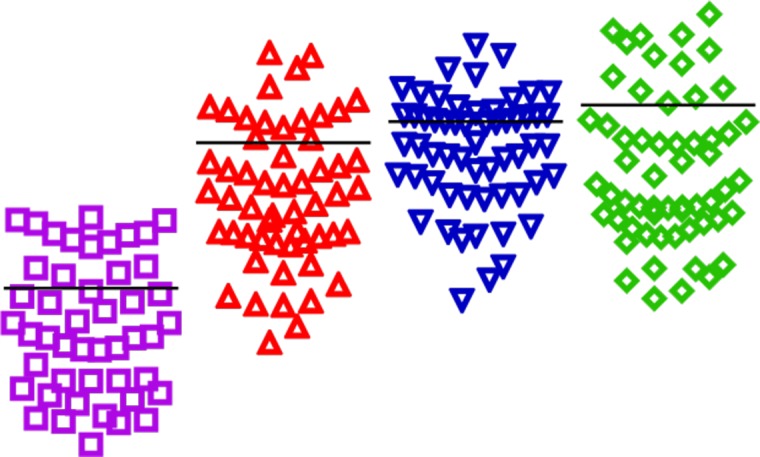
Expression of wt- ERα, ERαDel.2, ERαDel.4, ERαDel.7 and ERαDel.3, 4 in human endometrium in the total sample population (*n* = 58 patients) without taking into account the phase of the menstrual cycle or the existence of endometriosis Data from three to six experiments are expressed on a logarithmic scale in relation to GAPDH and ß-actin mRNA levels (mean ± standard deviation). ^*^p < 0.05, ^**^p < 0.01 in comparison with wt-ERa.

Q-PCR primers were designed to quantify the mRNA levels of wt-ERα and its four splice variants: Del.2, Del.4, Del.7 and Del.3,4. Our analyses showed that wt-ERα and all four splice variants were expressed in the human endometrium, both with and without endometriosis, and were all detectable throughout the menstrual cycle. Of all the ERα sub-types, wt-ERα was consistently expressed at the highest level, while Del.2 was expressed at the lowest levels. Del.4, Del.7, and Del.3,4 were expressed abundantly in the human endometrium. There was significantly stronger expression of wt-ERα compared to that of Del2 (*p* < 0.001), Del4 (*p* < 0.001), Del7 (*p* < 0.001), and Del3,4 (*p* < 0.001) within the total sample population (*n* = 58; Figure 3).

### Expression of ERα and its splice variants in human endometrium with and without endometriosis

**Figure 4:**
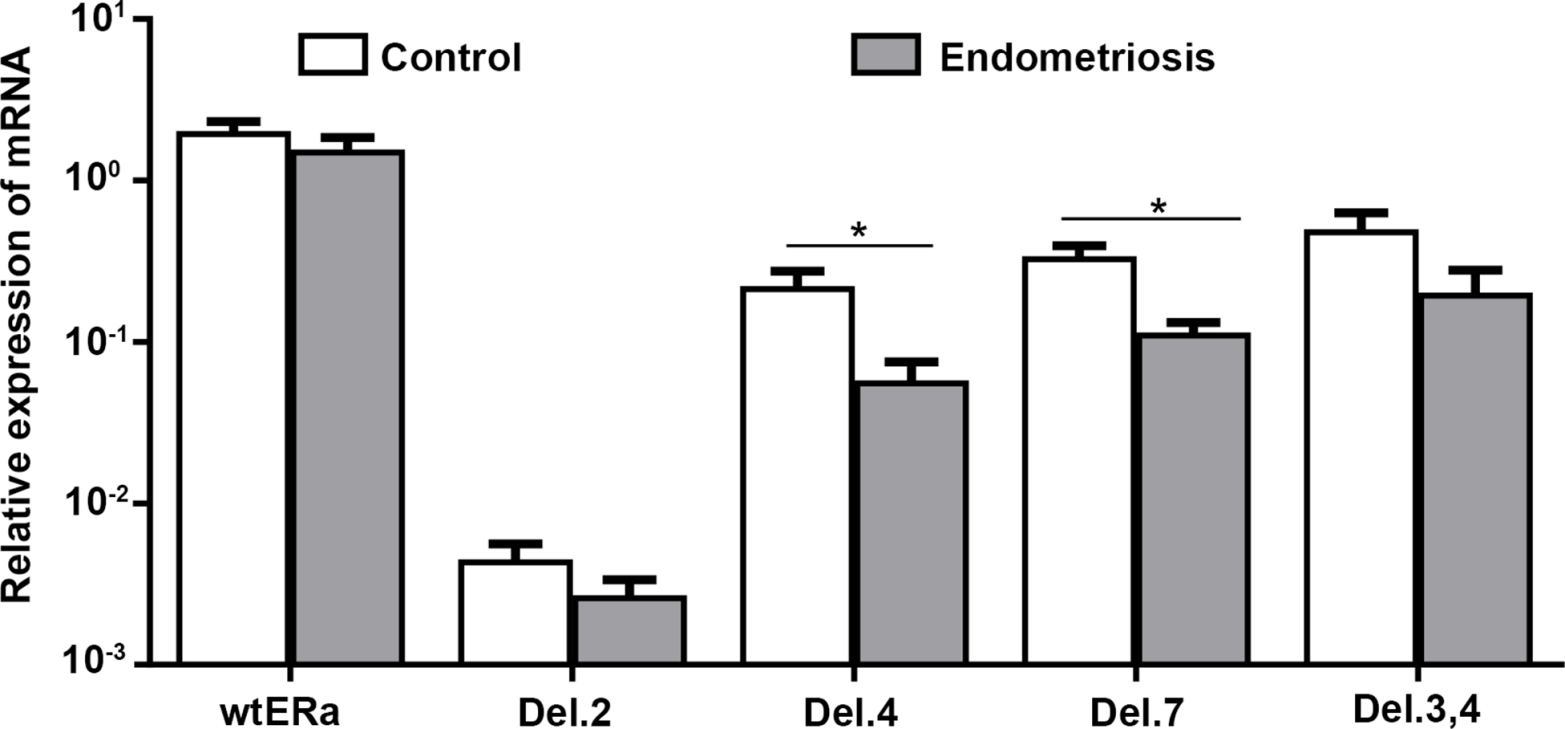
Expression of wt-ERα, ERαDel.2, ERαDel.4, ERαDel.7 and ERαDel.3,4 in patients with (*n* = 30) and without endometriosis (control, *n* = 28), without taking into account the phase of the menstrual cycle When cycle phase was ignored, the expression of ERαDel.4 and ERαDel.7 are significantly different between patients with and without endometriosis. Data from three to six experiments are expressed in logarithmic scale in relation to GAPDH mRNA levels (mean values ± standard deviation), ^*^p < 0.05, ^**^p < 0.01.

When the phase of the menstrual cycle was disregarded, there was a significantly diminished expression of Del.4 (*P* = 0.047) and Del.7 (*P* = 0.032) in the endometrium of patients with endometriosis compared with healthy endometrium. However, no significant difference was observed in terms of the expression of wt-ERα when compared between patients with and without endometriosis (Figure 4).

### Cycle-dependent expression of ERα in the human endometrium

Next, we compared the expression of wt-ERα and its splice variants in different phases of the menstrual cycle. These findings were independent of endometrial pathology (Figure 4) and were based on analysis involving control patients in the proliferative phase (*n* = 18), control patients in the secretory phase (*n* = 10), endometriosis patients in the proliferative phase (*n* = 22), and endometriosis patients in the secretory phase (*n* = 8).

**Figure 5:**
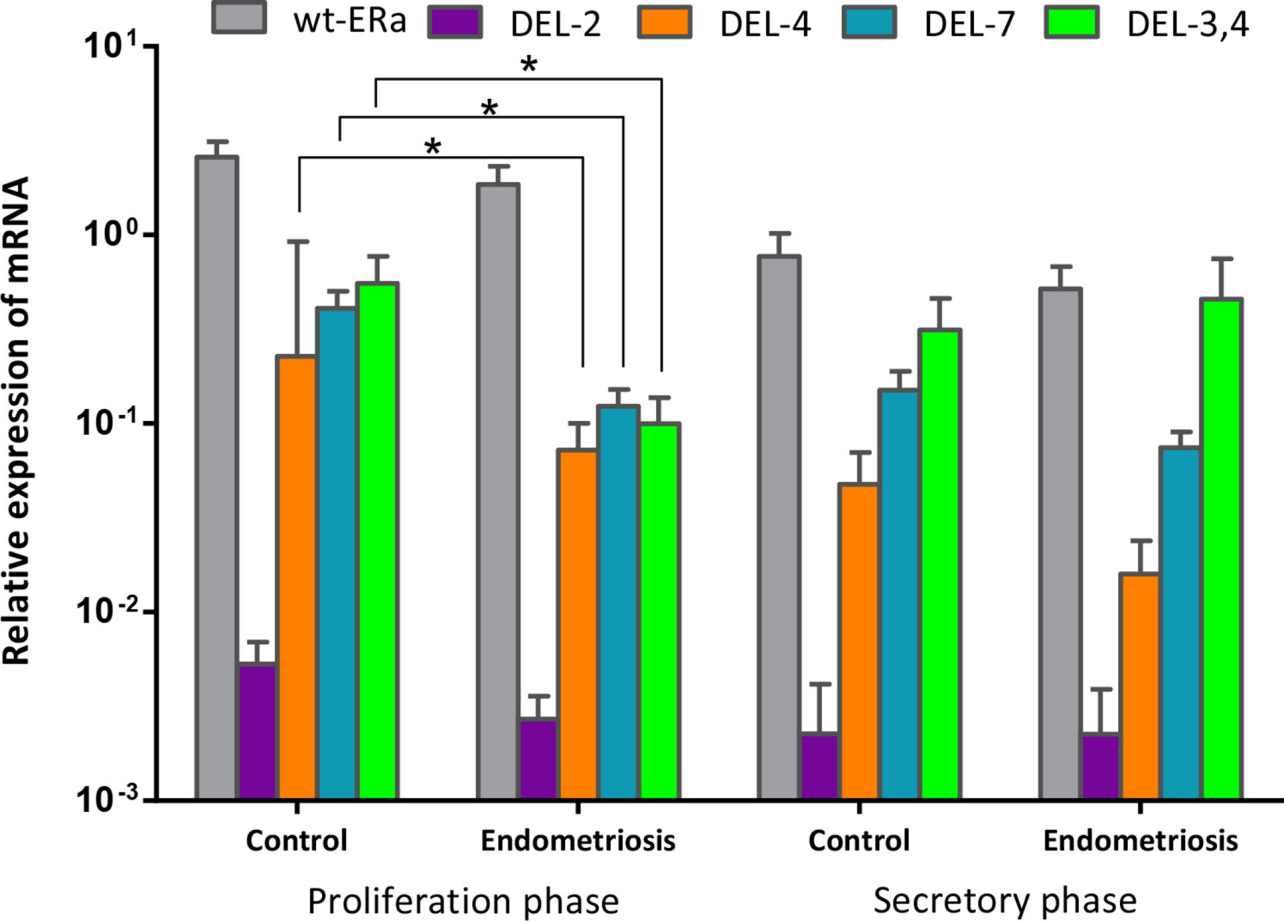
Cyclic expression of wt-ERα, Del.2, ERαDel.4, ERαDel.7 and ERαDel.3,4 in human endometrium The expression of wt-ERα and its splice variants were compared between four groups of patients characterized by cycle phase (proliferative and secretory phase) and the existence of endometriosis (healthy control patients vs. patients with endometriosis (control patients in the proliferative phase *n* = 18, control patients in the secretory phase *n* = 10, patients with endometriosis in the proliferative phase *n* = 22, patients with endometriosis in the secretory phase *n* = 8). Data from three to six experiments are expressed on a logarithmic scale in relation to GAPDH mRNA levels (mean ± standard deviation). ^*^p < 0.05, ^**^p < 0.01.

In the group of healthy women, the expression of ERα during the proliferative phase was significantly higher than that in the secretory phase (*p* = 0.021). Furthermore, during the proliferative phase, the expression of Del.4 (*p* = 0.014), Del.7 (*p* = 0.004) and Del.3,4 (*p* = 0.032) were significantly reduced in endometriosis patients compared with healthy patients. However, levels of ERα were not significant between the two groups (Figure 5).

### Ratio of wt-ERα to its splice variants

**Figure 6:**
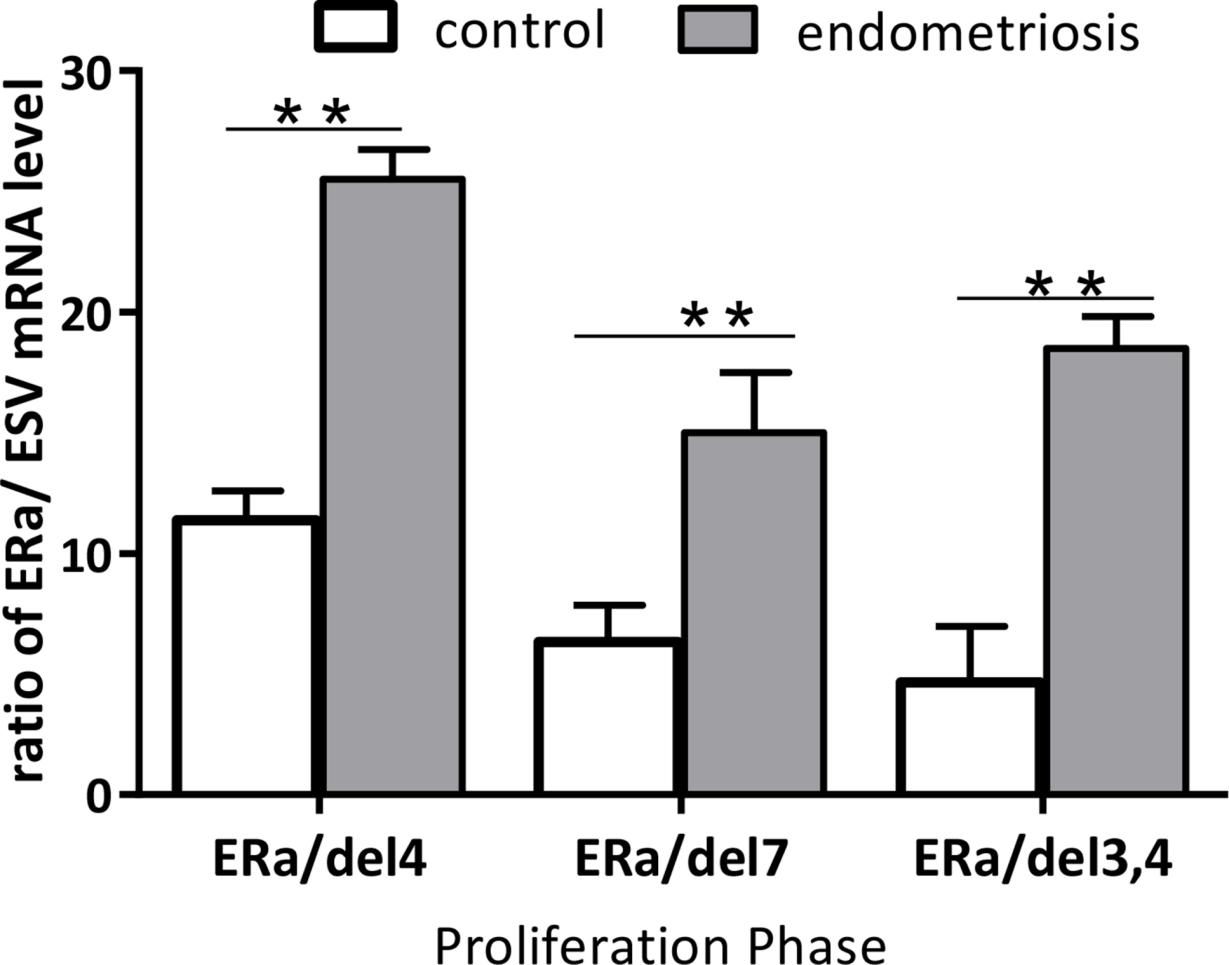
In the proliferative phase, the ratio of wt-ERα:ERα EL.4, wt-ERα:ERαDEL.7 and wt-ERα:ERαDEL.3,4 were significantly higher in endometriosis patients compared with the control group Data from three to six experiments were pooled and the ration of wt-ERα and its respective splice variants are shown as mean values ± standard deviation. ^*^p < 0.05, ^**^p < 0.01.

Here, we set out to determine how the ratio of wt-ERα and its splice variants varies in the human endometrium during different phases of the menstrual cycle in patients with and without endometriosis. We did this by analyzing levels of wt- ERα and ERα/del.2, ERα/del.4, ERα/del.7, and ERα/del.3,4 in proliferative and secretory phases and compared these results between patients with and without endometriosis. Only in the proliferative endometrium was the ratio between ERα and ERα/del.4 (*P* = 0.032), ERα/del.7 (*P* = 0.027), and ERα/del.3,4 (*P* = 0.035) significantly higher in endometriosis tissue than in the control group (Figure 6).

### Correlations between ERα and its splice variants with the severity of pain

As pain is the most classical clinical characteristic of endometriosis, we also aimed to investigate whether an increased ratio of ERα to its splice variants was correlated with the severity of pain in endometriosis.

**Figure 7:**
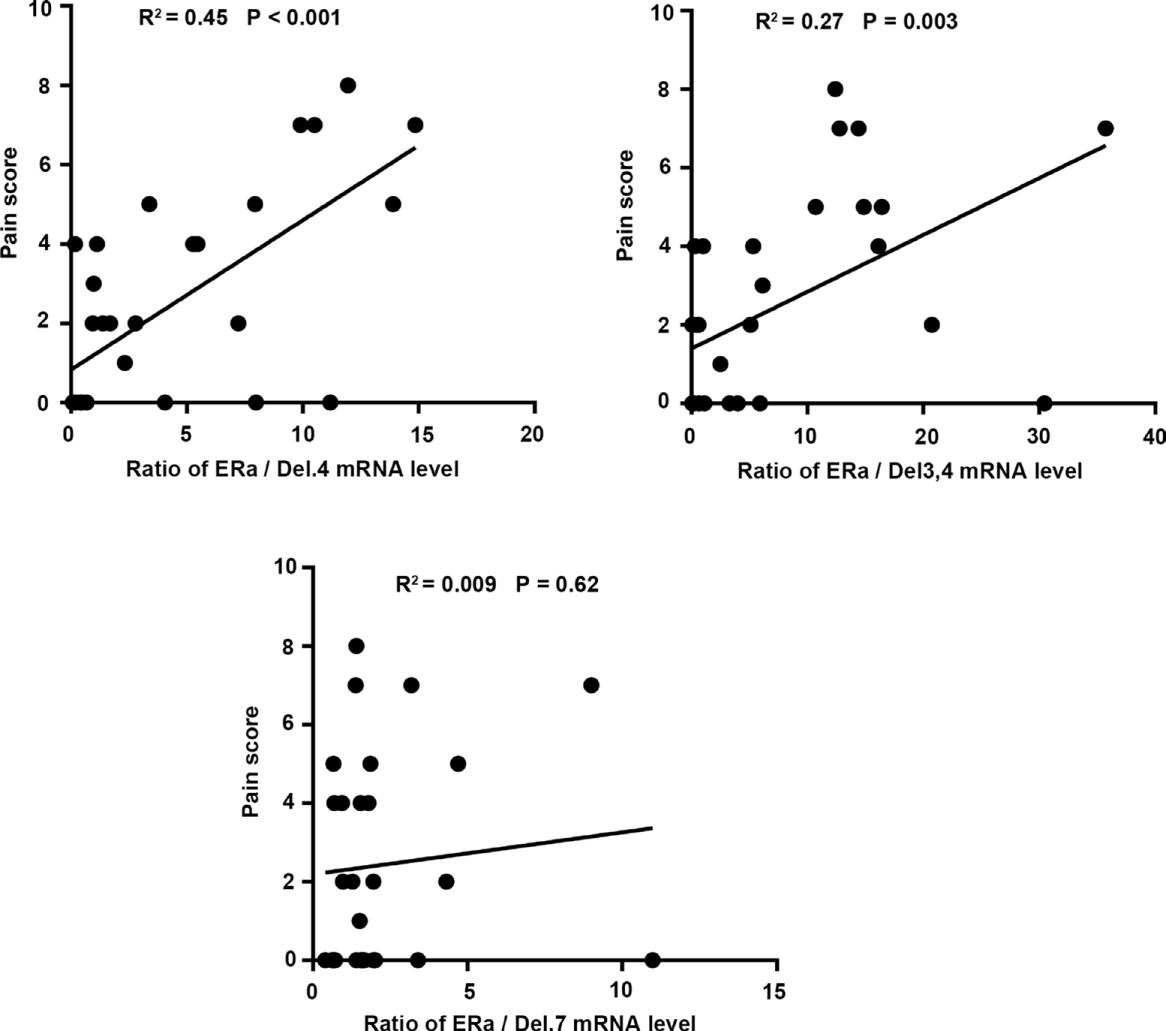
Correlation between the ratio of wt-ERα and its respective splice variants with dysmenorrhea pain score, which was assessed using the visual analogue scale (VAS) of 0–10 in endometriosis patients The ratios of wt-ERα:DEL.4 and wt-ERα:DEL.3,4 were positively correlated with the dysmenorrhea pain score (*r* = 0.61, *p* = 0.0003 and *r* = 0.54, *p* = 0.0023, respectively), although there was no significant correlation between the ratio of wt-ERα:ERα Del.7 and dysmenorrhea pain score.

Our analysis showed that the ratio of ERα:Del.4 and ERα:Del.3,4 in the proliferative phase were positively correlated with dysmenorrhea pain score (*r* = 0.61, *p* = 0.0003, *r* = 0.54, *p* = 0.0023, respectively). However, we did not find any significant correlation between the ratio of ERα:Del7 and the severity of dysmenorrhea (Figure 7).

## DISCUSSION

### ERα splice variants in the endometrium with and without endometriosis

Over the past decades, scholars of endometriosis have integrated several classical etiological models into a neoclassical perspective, which includes the notion that anatomical, genetic, epigenetic, and possibly environmental factors influence the risk of developing endometriosis [17, 18].

There has been considerable interest among researchers in targeting alternatively spliced estrogen receptors since they have been detected (along with wild type receptors) in virtually every estrogen-responsive tissue investigated, including the endometrium [19]. Owing to their presence in normal tissues, it has been speculated that alternatively-spliced variants are naturally occurring molecules, and consequently may exert biological function in the estrogen-induced signal transduction processes as either positive or negative modulators of gene expression [20, 21].

However, several questions remain unclear with regard to these splice variants. Firstly, prior to the present study, it was not known precisely which types of alternatively-spliced variants of the ER were expressed in the human endometrium. Secondly, we did not know whether alterations in the expression profile of these variants contributed to malignant transformation. Furthermore, it has not been established whether the relative expression of splice variants is related to the classical syndrome of endometriosis, especially in terms of the severity of dysmenorrhea.

To address some of the above questions, we used RT-PCR to identify several ERα splice variants in the human endometrium. WT-ERα was detected in all of the samples investigated, including those with and without endometriosis. Our data also showed that the ERαDel.2 variant was expressed at the lowest level of all variants, and in some samples was even undetectable, even by qPCR. This finding concurs with previous studies, which found minimal levels of ERαDel.2 in a range of other tissues and conditions, including patients with Alzheimer's disease (AD) [19, 22]. In contrast, the other ER splice variants, ERαDel.4, ERαDel.7 and ERαDel.3,4, were expressed at much higher levels.

### Characteristics of ERα splice variants

Functional studies in cell lines have provided evidence that the exon deletion variants detected in the present study (ERαDel.4, ERαDel.7 and ERαDel.2) are mainly localized in the cytoplasm [23–25]. Deletion of exon 4 suggests the loss of a portion of the ligand-binding domain and nuclear localization signal which explains the inability of ERαDel.4 mutants to bind to estradiol and to estrogen-responsive elements [23].

While the nuclear localization of this variant has also been described previously, ERαDel.4 is known to be loosely associated to the nuclear matrix and remains unaffected during hormonal treatment [23]. It is also worth noting that ERαDel.4 may also inhibit estrogen-independent transcriptional activity.

Deletion of exon 7 results in the deletion of 61 amino acids in the mature protein structure, indicating the loss of a significant portion of the hormone-binding domain and the ligand-binding domain with ligand-dependent trans-activation function (AF2) [26]. Consequently, ERα-Del.7 cannot bind ligands, is insensitive to estrogens and anti-estrogens, and cannot associate with coactivators.

The exon 7 deletion protein has been reported to bind DNA *in vivo* and has been reported to act as dominant-negative. Due to its ability to form heterodimers with both of the classical estrogen receptors, ERα and ERß, the ERαDel.7 variant results in a significant reduction of estrogen signaling in various cell types and may protect tissues from the effects of excessive estrogen.

High levels of ERαDel.7 in the endometrium, as found in our present study, concurs with previous literature describing the excessive expression of this isoform in the pituitary mammillary body, prefrontal cortex, lung, breast, and peripheral blood mononuclear cells [27–29]. However, little is known about the relative expression of the ERαDel.3,4 variant.

### ERα splice variants and endometriosis

Estrogens and steroid hormones play a primary function in the development and regulation of the female and male reproductive system. The classical signaling pathway involves estrogen receptors binding to ligands, translocating from the cytosol to the nucleus, dimerizing, recruiting other transcription modulating factors, and interacting with DNA promoter regions to influence transcription. The primary ligand for the human ER is E2, the predominant form of estrogen in premenopausal women.

For several decades, investigators have rigorously examined the role of ERs in the pathogenesis of disease. Much of this work has been conducted in the absence of specific attention to ER splice variants and how they might modify the physiological and pathological roles of E2 as a multifunctional tissue hormone. However, there is a growing body of evidence to suggest that some splice variants play an important role in both maintaining normal physiology and influencing susceptibility to disease. Compared with full-length “parent” receptors, splice variants differ in terms of the actions they mediate as a consequence of the lack of (or different configuration of) various receptor domains. Resultant splice variants may therefore fail to bind ligands, fail to translocate to the nucleus, fail to dimerize, or exhibit altered cofactor recruitment or the failure to bind and stimulate transcription at ERE or AP1 sites. If a splice variant fails to carry out these functions, it is unlikely that it will have any effect upon cell function. However, by failing to effectively perform one or two of these functions, inhibition of the full-length receptor may occur due to competitive ligand binding, heterodimer formation with the full-length formation receptor (rendering it inactive) and DNA binding without transcription (preventing the full length receptor from gaining appropriate access).

The function of ERα splice variants in the pathology of many diseases is not clearly defined. In most cases, this role is not yet fully understood and it is possible that ER splice variants could play a part. However, interestingly, it is possible that the dominant negative-function of ERα splice variants might protect tissues from excessive estrogenic signals. Furthermore, when the concentration of ERα splice variants declines in the endometrium, it is possible that the tissue may be exposed to more estrogenic signals. As we know, excessive estrogen plays an important role in the development and progression of endometriosis, which may help us to understand the role of ERα splice variants in the pathology of endometriosis.

### Ratio of wtERα to its splice variants and dysmenorrhea

The most common and most specific symptom of endometriosis is pain, typically in the form of progressive, secondary dysmenorrhea. This pain may also manifest as dyspareunia, dysuria, or dyschezia, or be referred to musculoskeletal regions, such as the flank or low back. The nature of the pain associated with endometriosis has been characterized inadequately, and the mechanisms involved remain unclear. Recent studies in both human and animal models suggest that the action of estrogen exacerbates pain sensitivity by stimulating the growth of a nerve supply (neurogenesis) [30], in parallel with the growth of new blood vessels (angiogenesis) into the ectopic endometrial tissue [30, 31].

Interestingly, in our present study, we observed high ratios of wt-ERα:ERαDel4, wt-ERα:ERαDel.7, and wt-ERα:ERαDel.3,4 in patients with endometriosis, which indicates that relatively more wt-ERα existed in endometriotic tissues and were thus exposed to more functional E2. Furthermore, the ratios of wt-ERα:ERαDel.4 and wt-ERα:ERαDel.3,4 were positively correlated with the severity of pain syndrome. In endometriosis patients, the relatively high ratio of wt-ERα to its associated splice variants, may provide an explanation for the promotion of both neurogenesis and angiogenesis and in the induction of dysmenorrhea.

### Summary

In summary, we identified a range of ERα variants in the human endometrium and compared the relative levels of expression of these splice variants in relation to the wt-ERα during different phases of the menstrual cycle. The specific expression of ERα-isoforms varied according to the phase of the menstrual cycle and whether the patient had endometriosis. The expression of ERα splice variants was significantly reduced in patients with endometriosis. Furthermore, the ration of wt-ERα to its splice variants was significantly higher in patients with endometriosis and correlated positively with the severity of pain.

Our present study may therefore provide a new direction for further studies in the pathology of endometriosis-associated pain, and may hold significant promise for the improvement of endometriosis targeted-treatment and medicine development.

## MATERIALS AND METHODS

A total of 58 women voluntarily participated in this study. Written consent was provided by all women and the study was approved by the Institutional Review Committee of the International Peace Maternity and Child Health Hospital, School of Medicine, Shanghai Jiao Tong University. None of the participants had pelvic inflammatory disease or cancer, and none had received any hormonal therapies during the 3 months leading up to the study.

The diagnosis of endometriosis was confirmed by visual impression at the time of surgery and histological examination of resected lesions. Endometriosis was scored and staged according to the revised classification system of the American Fertility Society (Society, 1985). All endometriosis patients were in severe stage, stage III–IV. Twenty-eight endometrium specimens were collected from healthy fertile women undergoing tubal sterilization and without any evidence of endometriosis at laparoscopy; these subjects formed our control group. Menstrual cycle phase was determined by the patient's last menstrual period and confirmed by histological dating of the endometrium. The intensity of pelvic pain symptoms just before surgery was assessed with a 10-cm visual analogue scale (VAS).

### RNA isolation

Total RNA was isolated from frozen endometrium samples using a Purelink RNA Mini Kit (ThermoFisher, Foster City, CA). All RNA was tested by spectrophotometric analysis on a Nanodrop 2000 (ThermoFisher, Foster City, CA) at absorbances of 260 nm and 280 nm, thus allowing calculation of the 260 nm:230 nm ratio. RNA was then reverse transcribed into cDNA using the Superscript IV First-Strand Synthesis System (ThermoFisher Scientific, Waltham, MA) in accordance with the manufacturer's instructions.

### Reverse transcription PCR (RT-PCR), cloning and DNA sequencing

RT- PCR was performed in 20 μl of PCR mixture comprising cDNA corresponding to 1 μg of total RNA, 1× PCR buffer, 0.2 mM dNTP mixture, and 0.2 M of forward and reverse primers, along with 0.25U of Q5 Hot Start High-Fidelity DNA polymerase (NEB, UK). PCR was performed with cycle reactions of 95°C for 5 s, 60°C for 30 s, and 72°C for 60 s, with an initial denaturing step of 95°C for 2 min and a final elongation step of 72°C for 2 min. The sequences of the oligonucleotide primers used for RT-PCR are shown in Supplementary Tables 1 and 2.

Amplified PCR products were then selected from 2% agarose gels and ER products characterized by cloning the gel-purified products into the pCR2.1-TOPO vector and DNA sequence analysis, as described previously (Majorbio Company, Shanghai, China).

### Real-Time PCR

Real-time PCR was carried out using a QuantiNova SYBR Green PCR kit (QIAGEN, Shanghai, China) in accordance with the manufacturer's instructions and by using an initial denaturation step at 95°C for 2 min, followed by 40 cycles with 10 s denaturation at 95°C and 30 s annealing at 60°C. PCR was carried out in an Applied Biosystems 7900 Real-Time PCR system (AB Applied Biosystems, Foster City, CA) by standard melting curve analysis. Negative controls were prepared by adding distilled water instead of the DNA template. The identity of the PCR products was verified by electrophoresis in 2% agarose gels and stained with 1 μl GelRed (Biosharp, Shanghai, China). After assessing molecular weight, each PCR product was purified using the Takara MiniBEST Agarose Gel DNA Extraction Kit v4.0 (Takara, Shiga, Japan), following the manufacturer's protocol, and finally verified by DNA sequencing (Majorbio Company, Shanghai, China).

In all RT-PCR experiments, a 142-bp GAPDH fragment was amplified as a reference housekeeping gene using the intron spanning primers, GAPDH-347 (GCACCGTCAAGGCTGAGAAC) and GAPDH-488 (ATGGTGGTGAAGACGCCAGT). Data were analyzed using the comparative ΔΔCT method, which calculates the difference between threshold cycle (CT) values of the target and reference genes from each sample and then compares the resulting ΔCT values between different samples.

### Statistical analysis

For descriptive statistics, data are presented as the mean ± standard deviation, and for categorical variables as counts or frequencies with percentages or proportions. The Student's *t*-test was used to evaluate differences between sample groups for normally distributed data, while the Mann–Whitney *U* test was used for non-parametric variables.

Correlations between the number of positive domains and symptom severity were assessed by Spearman's coefficient of rank correlation. All statistical assessments were two-tailed and were considered significant when *p* < 0.05. All data analysis was performed using SPSS (v16.0 for Windows) statistical software (SPSS Inc., Chicago, IL).

## SUPPLEMENTARY MATERIALS FIGURES AND TABLES


