# Supplementary material for: Reduced alternative splicing of estrogen receptor alpha in the endometrium of women with endometriosis
Source: Oncotarget. 2017 Nov 27;8(66):110176–86. doi: 10.18632/oncotarget.22701 (PMC5746374; doi:10.18632/oncotarget.22701)
Supplement: Supplementary file 1 [file oncotarget-08-110176-s001.pdf]

Reduced alternative splicing of estrogen receptor alpha in the endometrium of women with endometriosis

SUPPLEMENTARY MATERIALS

Supplementary Table 1: Primers for RT-PCR

Gene name	DNA Sequence
ERa-1–480BP-Forward	ATGCGCTGCGTCGCCTCTAA
ERa-1–480BP-Reverse	CTGCAGGAAAGGCGACAGCT
ERa-2–483BP-Forward	AACGCGCAGGTCTACGGTCA
ERa-2–483BP-Reverse	AATGGTGCACTGGTTGGTGG
ERa-3–487BP-Forward	ACGCCAGGGTGGCAGAGAAA
ERa-3–487BP-Reverse	CAAGGCACTGACCATCTGGT
ERa-4–546BP-Forward	GAGACATGAGAGCTGCCAAC
ERa-4–546BP-Reverse	GGGTGCTGGACAGAAATGTG
ERa-5–484BP-Forward	GGAGAGGAGTTTGTGTGCCT
ERa-5–484BP-Reverse	TGTGGGAGCCAGGGAGCTCT
ERa-6–732BP-Forward	CCCAGGCCAAATTCAGATAA
ERa-6–732BP-Reverse	CTGGAGTGTACACATTTCTGT

Supplementary Table 2: Primers for realtime-PCR

Name	Sequence
ERa-Del.2-Forward	CACTCAACAGCGTGTCTC
ERa-Del.2-Reverse	GTCGTTATGTCCTGTAGAATG
ERa-Del.4-Forward	TATGTGTCCAGCCACCAA
ERa-Del4-Reverse	ACAAAGCCACCTTTCATCA
ERa-Del3,4-Forward	CCAAGGAGACTCGCTACT
ERa-Del3,4-Reverse	CTAGAAGGTGGACCTGATC
ERa-Del7-Forward	GATGAATCTGCAGGGAGAGGAGT
ERa-Del7-Reverse	GCTCCATGCCTTTGTTACAGAATTA
